# Use of fiberoductoscopy for the management of patients with pathological nipple discharge: experience of a single center in Poland

**DOI:** 10.1007/s12282-018-0883-3

**Published:** 2018-06-25

**Authors:** Jacek Zielinski, Radoslaw Jaworski, Ninela Irga-Jaworska, Michal Pikula, Michael Hunerbein, Janusz Jaskiewicz

**Affiliations:** 1 Department of Surgical Oncology, Medical University of Gdansk, Smoluchowski Str 17, 80-214 Gdansk, Poland; 2 Department of Cardiac Surgery, Children’s Health Memorial Institute, Warsaw, Poland; 3 Department of Pediatrics, Hematology and Oncology, Medical University of Gdansk, Gdansk, Poland; 4 Department of Clinical Immunology and Transplantology, Medical University of Gdansk, Gdansk, Poland; 5 Department of General, Visceral and Cancer Surgery, Helios Klinikum Berlin-Buch, Berlin, Germany

**Keywords:** Fiberoductoscopy, Pathologic nipple discharge, Mammary duct lesions

## Abstract

**Background:**

Pathological nipple discharge (PND) is associated with serious clinical and diagnostic issues. Fiberoductoscopy (FDS) is a new diagnostic option in PND patients. This study summarizes our initial experience of FDS for the management of PND patients in a single center in Poland and assesses its safety.

**Methods:**

A total of 256 women with PND were included in this prospective, case-controlled, single-center study between 2006 and 2014. Of the 250 patients who underwent FDS, 154 had mammary duct lesions and 96 had no visible lesions. Subsequently, 129 patients with lesions identified by FDS underwent microductectomy and the lesions were pathologically evaluated.

**Results:**

The mean duration of FDS examination was 17 min. The most frequent intraductal lesion was amputation of a duct (35.1%), followed by circular narrowing or hyperplasia (22.7%). Final histological findings were unremarkable in 11.6% of cases, whereas mammary duct papilloma, duct ectasia, and ductal carcinoma in situ were detected in 71.3, 10.9, and 6.2% of cases, respectively.

**Conclusions:**

FDS is an innovative method for visualizing intraductal mammary lesions and allows accurate selection of mammary ducts with suspicious lesions that require surgical removal in women with PND.

## Introduction

Nipple discharge is associated with serious clinical/diagnostic issues and is categorized as physiological, para-physiological, and pathological [1]. Physiological nipple discharge may be related to lactation, whereas para-physiological nipple discharge may be caused by hypothyroidism, pituitary adenoma, ectopically produced prolactin, hypothalamic diseases, and pharmacotherapy. Pathological nipple discharge (PND) is unilateral, spontaneous discharge from a single mammary duct. The incidence of malignancy [ductal carcinoma in situ (DCIS) and invasive ductal carcinoma] in PND patients varies from 1 to 23% [2–6]. Classic diagnostic methods, such as galactography and ultrasonography, are not optimal for the differential diagnosis of PND, and consequently new diagnostic tools are being developed. Fiberoductoscopy (FDS) is a new diagnostic option in PND patients. This study summarizes our initial experience of FDS for the management of PND patients and assesses its safety.

## Materials and methods

**Fig. 1:**
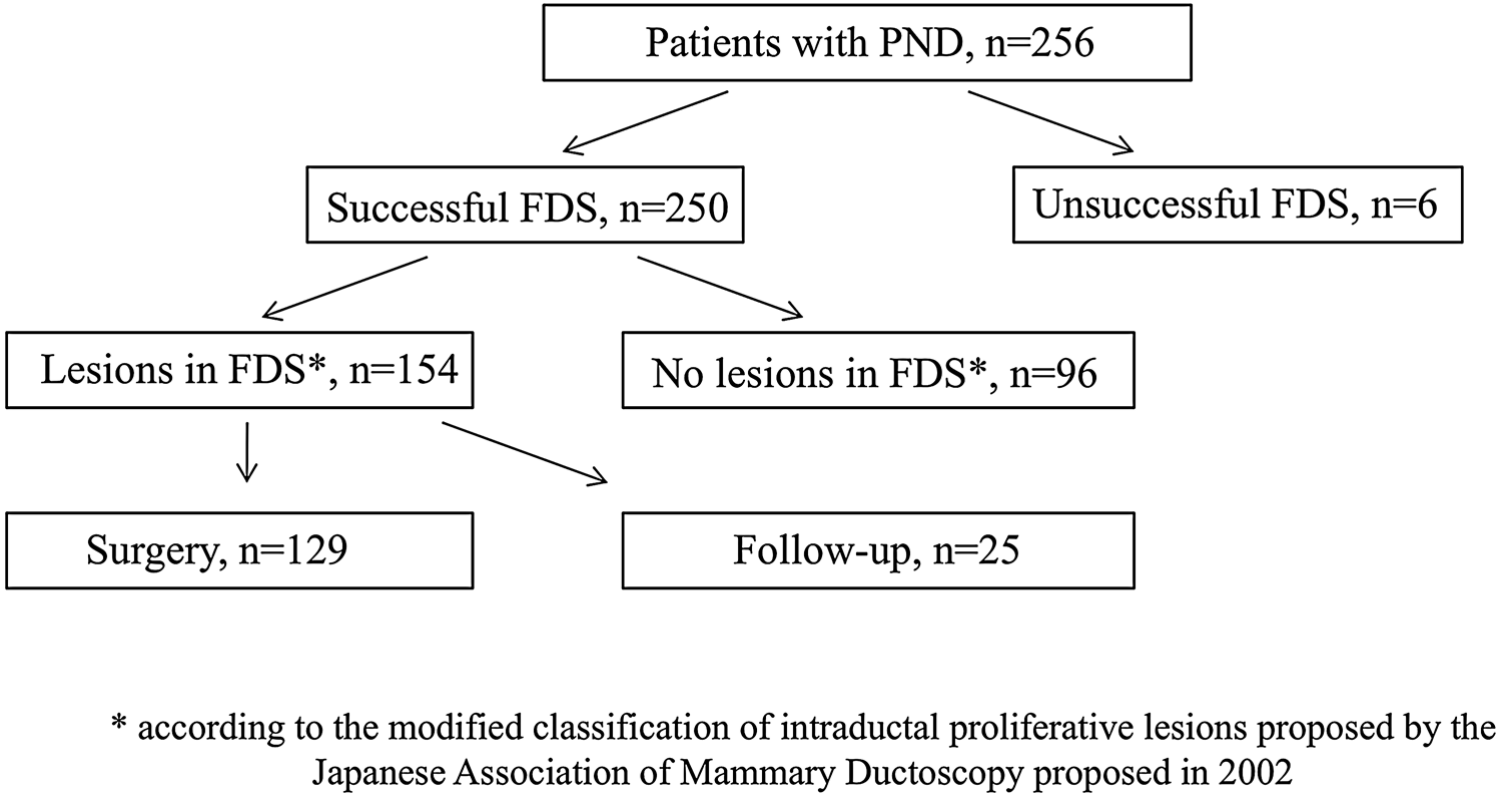
Flow chart of the study design. *PND* pathological nipple discharge, *FDS* fiberoductoscopy

This prospective, case-controlled, single-center study was performed according to the guidelines of the Ethics Examining Committee of Human Research at the Medical University of Gdansk, Poland (approval no. 466/2004). The subjects were PND patients treated at the Department of Surgical Oncology, Medical University of Gdansk, Poland, between 2004 and 2016. Women who had experienced PND for at least 1 month after receiving negative mammography and ultrasonography results for breast cancer and provided informed consent to participate in the study were included. Patients suspected of having breast cancer after mammography and/or ultrasonography, patients that were lactating, patients that had hypothyroidism, pituitary adenoma, ectopically produced prolactin, or hypothalamic diseases, and patients on antipsychotic, antihypertensive, antiemetic, or hormonal drugs were excluded. A total of 256 women were included in the study. Six patients did not undergo FDS due to unsuccessful cannulation. The remaining 250 patients were successfully cannulated, and mammary duct lesions were assessed by FDS. Of these, 154 patients had mammary duct lesions and 96 patients had no visible lesions in FDS examination. Subsequently, 129 patients with lesions detected by FDS underwent microductectomy after providing informed consent, while the other 25 patients were clinically observed (Fig. 1). In addition, 96 patients without visible lesions in FDS examination were clinically observed.

**Fig. 2:**
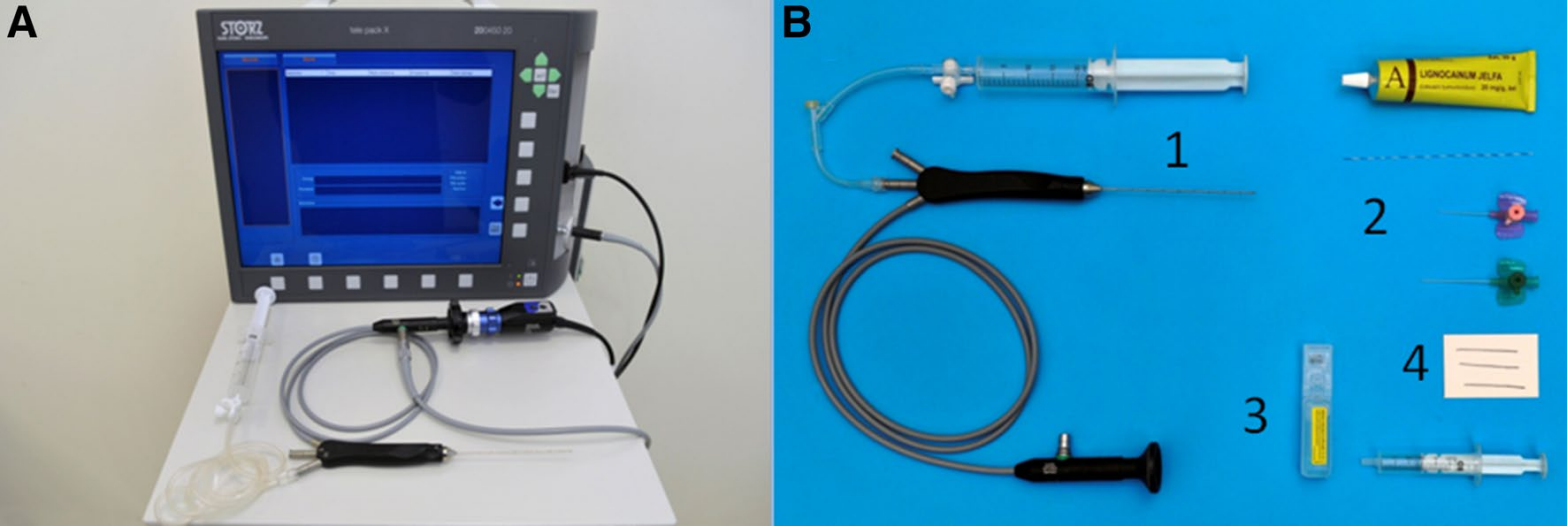
**a** Fiberoductoscopy system. **b** Fiberoductoscopy was performed using (1) a diagnostic cannula, (2) dilators, (3) a cytology set, and (4) sutures to mark the mammary duct for microductectomy (Storz, Germany)

FDS was performed using a fiberoductoscope (Volpi AG, Switzerland, or Storz, Germany), a light source, a camera (Camera Handpiece, Switzerland), and an image monitor and recorder (PC equipped with Total Media software, ArcSoft Inc., USA). The outer diameter of the fiberoductoscope was 0.45–1.1 mm, and its maximum exploratory length was 120 mm (Fig. 2). The fiberoductoscope was inserted and FDS was performed by one surgeon with assistance. At 15 min prior to examination, local anesthesia was applied to the nipple in the form of lidocaine as an aerosol (Egis Pharmaceuticals Ltd., Hungary) or lidocaine and prilocaine as anointment (AstraZeneca Pharma, Poland). Although patients with spontaneous PND already had a somewhat dilated nipple orifice, dilators were introduced into the nipple orifice to dilate the ostium of the lactiferous ducts. The fiberoductoscope was then gently introduced into the nipple orifice. The major lactiferous ducts and segmental branches were visualized, and the presence of any papillary lesions was recorded. The depths of mammary duct lesions were also noted. The marking sutures were left to guide microductectomy.

Lesions in mammary ducts were assessed according to the modified classification of intraductal proliferative lesions proposed by the Japanese Association of Mammary Ductoscopy in 2002 with our own modifications. The following types of intraductal proliferative lesions were recorded: single papilloma, multiple papilloma, amputation of a duct, circular narrowing or hyperplasia, duct ectasia, ambiguous results (i.e., reddening or red spots), and microcalcification [7, 8]. Lesions detected by FDS were removed via microductectomy and pathologically evaluated.

The learning curve after introduction of FDS at our center was assessed. To this end, the percentage of successful mammary duct cannulations and the duration of FDS examination were recorded. Moreover, all patients were carefully observed during and after FDS, and mammary duct injury, local inflammation, redness in the vicinity of the nipple, and pain were noted. Follow-up analysis was performed 6 months after FDS in all patients. The recurrence rate of PND in patients who underwent microductectomy was calculated.

### Statistics

For continuous variables, the mean, standard deviation (SD), and range are provided. Data for categorical variables were compared using Pearson’s chi-squared test, and Yates’ correction and Fisher’s exact test were applied if the number of sub-groups was low. Statistical analyses were conducted using SPSS version 13.0 (SPSS Inc., USA).

## Results

Among the 256 women who qualified for this study, six did not undergo FDS due to unsuccessful cannulation caused by deformation of the nipple or a narrow orifice. All unsuccessful cannulations occurred during the first 3 years of the study; the cannulation success rate was 91.5% in this period compared with 100% in the other years (*p* < 0.001). After FDS had been performed in the first 58 patients, all subsequent cannulations were successful.

**Fig. 3:**
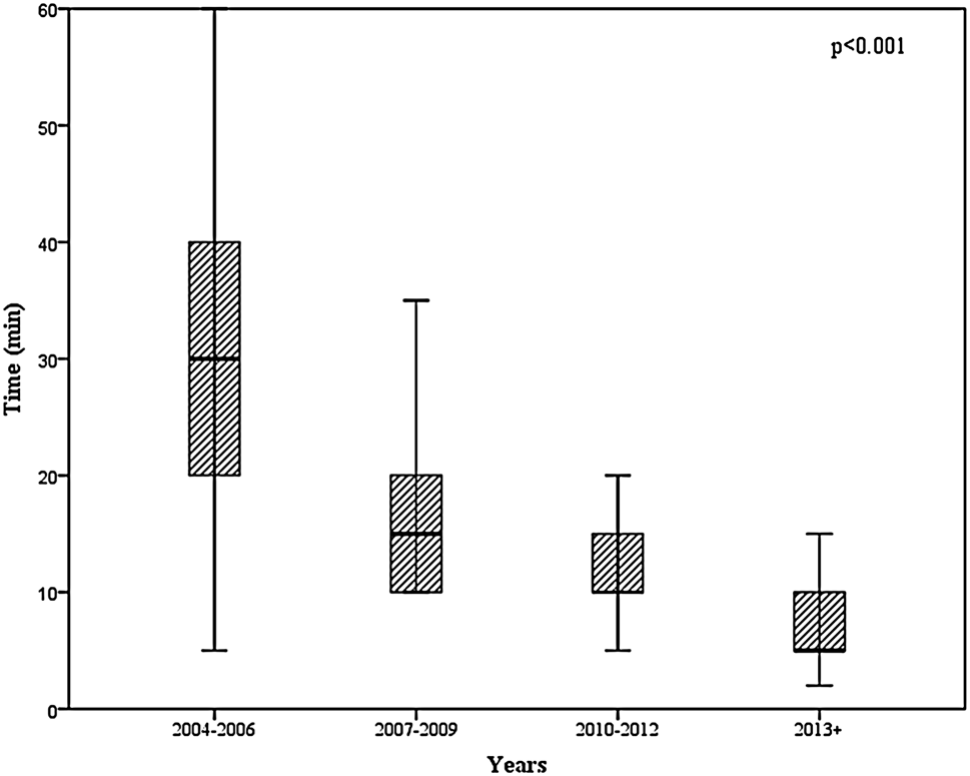
Duration of the fiberoductoscopy examination in consecutive years after its implementation

Mammary duct cannulation was successful in 250 patients. Of these, FDS detected mammary duct lesions in 154 patients, whereas there were no visible lesions in the other 96 patients. The mean age, weight, and body mass index of patients who underwent FDS were 51 years (SD 13 years; range 21–84 years), 70 kg (SD 14 kg; range 49–125 kg), and 26 kg/m^2^ (SD 5 kg/m^2^; range 17–41 kg/m^2^), respectively. Nipple discharge was bloody and serous in 136 (54.4%) and 114 (45.6%) patients, respectively. The mean duration of FDS examination was 17 min (SD 12 min; range 2–65 min) in all patients and varied significantly between the four time periods; it was 31 min (SD 13.4 min; range 5–65 min), 17 min (SD 7.8 min; range 10–40 min), 12 min (SD 3.6 min; range 5–30 min), and 7 min (SD 2.8 min; range 2–15 min) in 2004–2006, 2007–2009, 2010–2012, and 2013–2016, respectively (*p* < 0.001) (Fig. 3).

Mammary duct lesions were observed in 154 patients by FDS. Lesions were usually located 20–39 mm from the lactiferous ducts of the nipple (Table 1). The detailed characteristics of the lesions assessed by FDS are presented in Table 1. The most frequent intraductal lesion was amputation of a duct (35.1%), followed by circular narrowing or hyperplasia (22.7%).

**Table 1:** Characteristics of intraductal lesions assessed by fiberoductoscopy in 154 patients with pathological nipple discharge

Depth of IPLs (mm)	
0–9	4 (2.6%)
10–19	26 (16.9%)
20–39	104 (67.5%)
≥ 40	20 (13%)
Type of IPLs^a^	
Single papilloma	12 (7.8%)
Multiple papilloma	14 (9.1%)
Amputation of a duct	54 (35.1%)
Circular narrowing or hyperplasia	35 (22.7%)
Duct ectasia	14 (9.1%)
Ambiguous results (reddening, red spots)	21 (13.6%)
Microcalcification	4 (2.6%)

^a^Based on the modified classification of intraductal proliferative lesions proposed by the Japanese Association of Mammary Ductoscopy in 2002.[7, 8]

*IPL* Intraductal proliferative lesion

The FDS results could be compared with post-operative histopathologica findings in 129 of 154 patients who qualified for surgical treatment (83.8%). The other 25 women did not consent to surgical excision and were followed up; these patients had duct ectasia or ambiguous results. The final histological findings of the ductal excision were unremarkable in 15 cases (11.6%) and suspicious in 114 cases (88.4%). Mammary duct papilloma, duct ectasia, and DCIS were found in 92(71.3%), 14 (10.9%), and 8 (6.2%) cases, respectively. There were significant associations between the type of discharge and the final pathologic findings (Table 2); all patients with DCIS had bloody discharge.

**Table 2:** Associations between pathological nipple discharge type and final pathologic findings

Pathologic findings	Type of discharge		*P* value
	Serous (*n* = 54) (%)	Bloody (*n* = 75) (%)	
Papilloma (*n* = 92)	37 (40.2)	55 (59.8)	0.001
DCIS (*n* = 8)	0	8 (100)	
Duct ectasia (*n* = 14)	11 (78.6)	3 (21.4)	

*DCIS* ductal carcinoma in situ

### Safety and complications of FDS

Only 8 (3.2%) of the 250 patients who underwent FDS suffered complications. Local injury of mammary ducts was detected during FDS in two patients (0.8%) in the first 3 years after the introduction of this procedure at our center. Local inflammation (redness in the vicinity of the nipple and pain) in the region of cannulation was detected during the follow-up in four patients (1.6%); however, infection was not confirmed in any patient. PND recurred in 2 of 129 (1.6%) patients who underwent microductectomy. Both these patients required further surgery to remove more mammary tissue. None of the other patients experienced PND recurrence or a change in their quality of life.

## Discussion

PND constitutes a crucial issue among patients with breast diseases for epidemiological, diagnostic, and therapeutic reasons. Galactography is currently considered the state-of-the-art approach for diagnosing mammary intraductal lesions; however, this technique cannot assess duct narrowing in detail [9, 10]. In a study by Ohlinger et al., galactography achieved in this issue a sensitivity and specificity of 81.3 and 44.4%, respectively [10]. It is difficult to detect intraductal processes in mammograms; sensitivity and specificity are 9–57.1 and 33.3–100%, respectively [10–12]. The low sensitivity reported by Albrecht et al. was explained by the large number of false-negative results [12]. PND is often overlooked as an indication of DCIS. The incidence of malignancy (DCIS and invasive ductal carcinoma) in PND patients varies from 1 to 23% [2–6]. Consistent with the study by Fisher et al. [13], nipple discharge was bloody and serous in 54.4 and 45.6% of patients in the current study, respectively, and all patients with DCIS had bloody nipple discharge.

There is no consensus regarding how to diagnose PND patients; however, surgery involving selective duct excision (microductectomy) or more extensive tissue excision (segmentectomy and quadrantectomy) is considered standard [14, 15]. All surgical procedures are invasive and carry the risk of complications [16]. In our opinion, the diagnosis and treatment of PND could be improved using a technique to directly visualize mammary ducts. FDS is a minimally invasive procedure that visualizes the ductal epithelium of the breast via the nipple [17]. Due to the high quality of the optical system and the small diameter of the diagnostic cannula, FDS enables direct visualization of mammary ducts from lactiferous on the nipple to the subsegmental ducts [18, 19]. This procedure can be performed under local anesthesia in out-patient clinics [20]. The use of FDS as part of standard medical care for PND patients is limited to a few medical centers worldwide. FDS has evolved over recent decades, and additional techniques have been developed. However, reports on FDS remain limited and most clinicians are unfamiliar with this technique. Despite this, FDS is a useful technique for evaluating and treating PND [14, 20].

The cannulation success rate and duration of examination are important attributes of the FDS procedure, but are seldom described in the literature. Zagouri et al. investigated how clinicians can master FDS by performing this technique ex vivo on breasts removed via mastectomy [21]. They estimated that clinicians need to perform at least 20 examinations to achieve a cannulation success rate of 90%. In the current study, all cannulations were successful after 58 examinations had been conducted. However, learning of the FDS technique may be connected with complications. We recorded mammary duct injuries during FDS examination and local inflammatory/pain at the site of cannulation during the follow-up in 0.8 and 1.6% of patients, respectively. The duration of FDS examination is connected with how well the clinician has mastered the cannulation technique. The mean duration of FDS examination gradually decreased over the course of this study, from 31 min in 2004–2006 to 7 min in 2013–2016.

FDS analysis demonstrated that lesions were mostly located at a depth of 20–39 mm (67.5% of patients). The abovementioned scope of depth of introducing the device indicates that the intraductal proliferative lesions were located between the lactiferous sinus and the subsegmental ducts. Similarly, Denewer et al. most frequently found lesions at a depth of 20–40 mm (44.4%), followed by 40–60 mm (33.3%) [22]. The use of thinner devices would likely enable visualization of the majority of subsegmental ducts, and this may facilitate the detection of cancers in breast ducts located at the ductolobular border. FDS also enables mammary ducts to be accurately marked for minimally invasive surgical resection of breast tissue, as demonstrated in this study. Khan et al. confirmed that office FDS allows the accurate and safe selection of women with nipple discharge who require surgery [23].

In the current study, intraductal lesions diagnosed by FDS were mostly amputation of a duct (35.1%) and circular narrowing or hyperplasia (22.7%), while pathological examination detected papilloma in 71.3% of patients. Of the 129 patients who underwent surgery, eight were diagnosed with DCIS, all of whom had bloody nipple discharge. In the study by Fisher et al., the most common lesion observed by FDS was papilloma, followed by duct ectasia, and the percentage of patients with DCIS (6%) was similar to that in the current study (6.2%) [13].

FDS has been reported to have a sensitivity of 53.2–71.2% and a specificity of 49.4–61.5% for diagnosis of PND [10–12]. We previously found that FDS has a sensitivity of 68.1%, a specificity of 77.3%, a positive predictive value of 90.4%, and a negative predictive value of 44.1% [24]. Waaijer et al. reported that FDS has a pooled sensitivity and specificity for suspicious findings of 50 and 83%, respectively, based on a meta-analysis [14]. FDS is a good option for diagnosis of PND and can be used to guide minimally invasive surgical removal of mammary tissue. However, as highlighted in a review article by Waaijer et al., although FDS detects about 94% of all underlying malignancies in PND patients, it cannot reliably discriminate between malignant and benign findings [14]. The introduction of transductal intervention devices may facilitate the use of FDS for the treatment as well as the diagnosis of PND patients [25, 26]. Further development of FDS may reduce the rate of surgical excision in patients with benign lesions. This is hypothesized to reduce hospital costs by avoiding unnecessary invasive procedures; however, a cost–benefit analysis is required to confirm this [14].

FDS is an innovative method for visualizing intraductal mammary lesions and allows accurate selection of mammary ducts with suspicious lesions that require surgical removal in women with nipple discharge. This procedure can be performed under local anesthesia in out-patient clinics. The introduction of FDS in our clinic has improved the diagnosis of PND patients. In particular, patients with bloody nipple discharge can be screened for early stages of breast cancer using FDS. The diagnostic features of FDS need to be further investigated, and the procedure should be improved to enable direct biopsy or excision of intraductal lesions.
